# Two-step production of anti-inflammatory soluble factor by *Lactobacillus reuteri* CRL 1098

**DOI:** 10.1371/journal.pone.0200426

**Published:** 2018-07-06

**Authors:** Milagros Griet, Graciela Font de Valdez, Carla L. Gerez, Ana V. Rodríguez

**Affiliations:** Centro de Referencia para Lactobacilos (CERELA), Consejo Nacional de Investigaciones Científicas y Técnicas (CONICET), San Miguel de Tucumán, Tucumán, Argentina; Laurentian University, CANADA

## Abstract

We have demonstrated previously that a soluble factor (LrS) produced by *Lactobacillus* (*L*.) *reuteri* CRL 1098 modulates the inflammatory response triggered by lipopolysaccharide. In this study, the production of LrS by *L*. *reuteri* CRL 1098 was realized through two steps: i) bacterial biomass production, ii) LrS production, where the bacterial biomass was able to live but did not proliferate. Therefore, the simultaneous evaluation of the effect of different factors on the growth and LrS production was performed. Biomass production was found to be dependent mainly on culture medium, while LrS production with anti-inflammatory activity depended on culture conditions of the biomass such as pH, agitation and growth phase. The *L*. *reuteri* CRL 1098 biomass and LrS production in the optimized culture media designed for this work reduced the complete process cost by approximately 95%, respectively to laboratory scale cost.

## Introduction

In the past decades, several worksdemonstrated the capacity of probiotic lactic acid bacteria (LAB) to modulate immune response. In this way, some strains may have prophylactic and therapeutic effect in different complex disorders, including inflammatory diseases [1–4]. In addition, increased research reports revealed that certain surface-associated and extracellular components produced by probiotic bacteria could be responsible for some of their beneficial effects: they include exopolysaccharides, bacteriocins, surface-associated and extracellular proteins and peptides and lipoteichoic acid [5–9].

The current demand for new products enriched with probiotics or its functional extracellular componentsrequires new economic media for their production at an industrial scale [10]. In probiotic product development processes, microbial biomass and bioactive metabolites areconsidered raw material. Thus, the cost of the culture media for the industrial production of both is a determinant factor [11]. To produce significant biomass, Lactobacilli have complex nutrient requirementssuch as amino acids, yeast extract, carbohydrates, vitamins and minerals [12]. However, the use of these nutrient supplements in large quantities is very expensive [13]. Therefore, a development of new culture media taking into account nutrient requirements and growth parameters could lead to a more economical probiotic production [14]. Statistical methodology is efficiently used to optimize several bioprocesses such as the development of a simplified, and hence more economical, culture medium, for biomass production [14–15]. In this situation, the fractional factorial designs have been successfully applied for studying the effects and interactions of distinct nutrients on *Lactobacillus* growth [15–17].

We have demonstrated previously, that *L*. *reuteri* CRL1098 can produce a supernatant (LrS) able to reduce the TNF-α production by human peripheral blood mononuclear cells (PBMC) [18]. Bearing in mind that TNF-α levels reduction is an effective treatment for inflammatory diseases, we further investigated whether LrS was able to modulate the inflammatory response used different models. For this, we used *in vitro* and *in vivo* models. Results showed that LrS exerted anti-inflammatory actions in LPS-challenged murine macrophages model and in acute lung injury induced by LPS in mice [19]. The compound present in LrS, responsible for the anti-inflammatory actions, is a peptide moiety component. Using RP-HPLC, this peptide was isolated keeping of the capacity to reduce the TNF-α production by PBMC. The peptide was identified by mass spectrometry as 5785 Da [18], and its sequence analysis is currently in progress.

**Fig 1:**
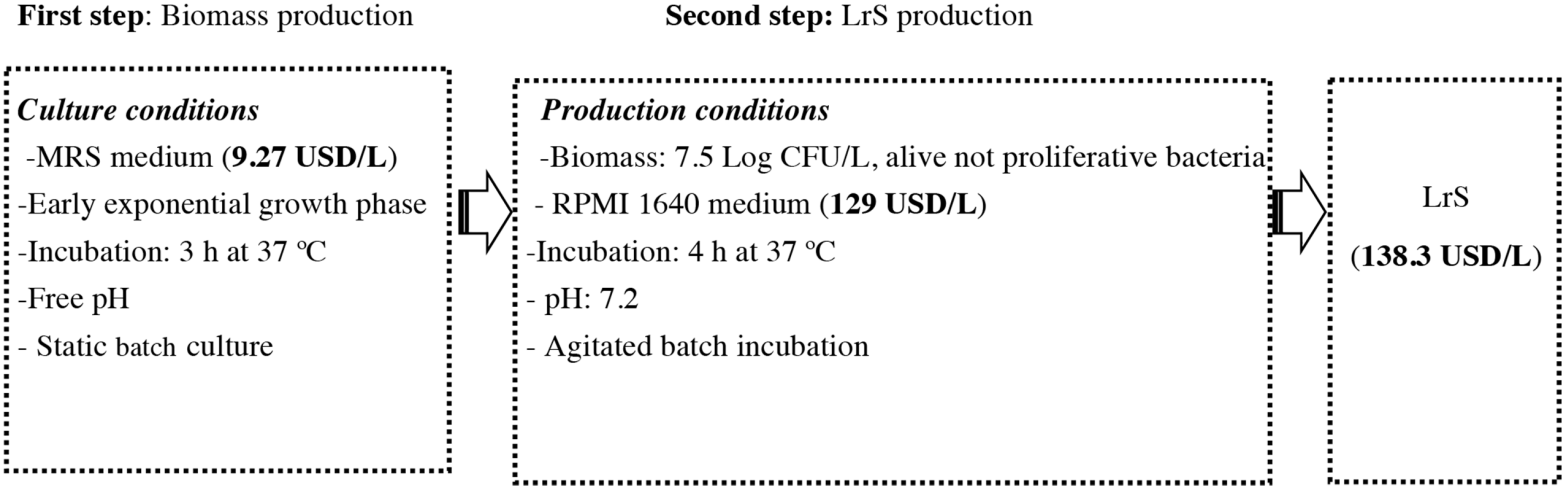
Process for obtaining of anti-inflammatory-soluble factor (LrS) from *L*. *reuteri*CRL 1098 at laboratory scale.

At laboratory scale, the production of LrS involves two steps: production of *L*. *reuteri* CRL1098 biomass until the early exponential growth phase in MRS broth [20], and then the transfer of the obtained biomass to RPMI 1640 medium (GIBCO, Grand Island, NY, USA) with incubation of 4 h, where the bacteria are able tolive but do not proliferate and produce LrS (Fig 1). Some preliminary assays revealed that the compound is not associated with the growth of *L*. *reuteri* and the extension of the incubation time in the second step does not increase the anti-inflammatory activity of LrS (unpublished data).

The cost analysis of LrS production process reveals that MRS and RPMI 1640 media are very expensive and they significantly increase the cost of production (LrS: 138.3 USD/L). Therefore, the aims of this study were to formulate low-cost media for (i) *L*. *reuteri* CRL1098 biomass production (first step) using a fractional factorial design and (ii) LrS production (second step). Fermentation parameters such as pH, culture agitation and bacterial growth phase were also determined.

## Materials and methods

### Bacterial growth

*L*. *reuteri* CRL1098, an anti-inflammatory soluble factor producing strain, was provided by the Culture Collection of Centro de Referencia para Lactobacilos, CERELA. Tucumán, Argentina. Stock cultures were maintained at −20 °C in 10% reconstituted skim milk supplemented with 0.5% yeast extract, 1.0% glucose, and 10.0% glycerol (w/v). Prior to experimental use, cultures were propagated (1%, v/v) in MRS medium containing per liter: 10 g meat peptone, 10 g meat extract, 5 g yeast extract, 3 g sodium acetate, 2 g K_2_HPO_4_, 2 g ammonium citrate, 20 g glucose, 1 mL Tween 80, 0.2 g MgSO_4_.7H_2_O and 0.038 g MnSO_4_.H_2_O, final pH 6.5 [20], and incubated at 37 °C for 18h without agitation.

### First step: Design of a low-cost culture medium for *L*. *reuteri* CRL1098 biomass production

The 2^10−6^ fractional factorial design was used to select significant culture medium components affecting the *L*. *reuteri* CRL1098 biomass production. Design Expert version 7.0 statistical software (Stat-Ease, Minneapolis, MN) was used for the experimental design and regression analysis of the experimental data. The MRS culture medium was used as the base culture medium for the growth of *L*. *reuteri*. The factors and levels taken into consideration were: meat peptone (0.0, 0.5 and 1.0%), meat extract (0.0, 0.5 and 1.0%), yeast extract (0.0, 0.25 and 0.5%), sodium acetate (0.0, 0.15 and 0.3%), K_2_HPO_4_ (0.0, 0.1 and 0.2%), ammonium citrate (0.0, 0.1 and 0.2%), glucose (0.5, 1.25 and 2.0%), Tween 80 (0.0, 0.05 and 0.1%), MgSO_4_.7H_2_O (0.0, 0.01 and 0.02%), MnSO_4_.H_2_O (0.0, 0.0019 and 0.0038%). A 2^10−6^ fractional factorial design was utilized to screen 38 different culture media defined in 2 blocks with three central points in each block (Table 1). The batch fermentations were carried out in 100 mLPyrex bottles containing 80 mL of fermentation medium. The different culture media were inoculated (2% v/v) with *L*. *reuteri* CRL1098 and incubated at 37°C for 3h (exponential growth phase) without agitation. The responses evaluated were LAB growth and pH changes (pHmeter PT-10 model, Sartorius AG, Goettingen, Germany). Cell viability was determined by plating appropriate dilutions of the cultures in MRS agar (MRS Britania, Buenos Aires, Argentina, plus 15 g/L agar). Plates were incubated at 37°C for 48h and the colony-forming units (CFU/mL) were determined.

**Table 1:** Fractional factorial design and results of *L*. *reuteri* CRL1098 biomass production, ΔpH and cost of different growth medium.

Culturemedium	Factors (%)										Growth (Log CFU/mL)	ΔpH^♯^	Cost (USD/L)
	MP	ME	YE	SA	K	AC	G	T	Mg	Mn			
**BLOCK 1**													
**1**	1	1	0	0	0	0.2	2	0.1	0	0	7.76	0.43	8.32
**2**	1	1	0	0.3	0.2	0	0.5	0.1	0	0	7.96	0.65	7.76
**3**	0	0	0	0.3	0	0	2	0	0	0	7.02	0.39	0.96
**4**	0	0	0.5	0	0	0	0.5	0.1	0	0	7.13	1.62	0.81
**5**	0	0	0.5	0	0.2	0	2	0	0.02	0.0038	7.27	0.75	1.59
**6**	1	1	0.5	0.3	0	0.2	0.5	0	0	0	7.70	0.37	8.43
**7**	1	1	0.5	0	0	0	0.5	0.1	0.02	0.0038	7.98	1.23	7.98
**8**	0	0	0.5	0.3	0	0.2	0.5	0	0.02	0.0038	7.06	0.24	1.27
**9**	1	1	0	0	0.2	0.2	0.5	0	0.02	0.0038	7.66	0.46	7.90
10*	0.5	0.5	0.25	0.15	0.1	0.1	1.25	0.05	0.01	0.0019	7.69	0.54	4.74
11*	0.5	0.5	0.25	0.15	0.1	0.1	1.25	0.05	0.01	0.0019	7.70	0.60	4.74
**12**	1	1	0.5	0	0.2	0	2	0	0	0	7.54	0.55	8.75
**13**	0	0	0.5	0.3	0.2	0.2	2	0.1	0	0	7.24	0.33	2.10
14*	0.5	0.5	0.25	0.15	0.1	0.1	1.25	0.05	0.01	0.0019	7.74	0.68	4.74
**15**	0	0	0	0	0.2	0.2	0.5	0	0	0	7.02	0.26	0.73
**16**	0	0	0	0	0	0.2	2	0.1	0.02	0.0038	7.34	0.14	1.16
**17**	0	0	0	0.3	0.2	0	0.5	0.1	0.02	0.0038	7.13	0.32	0.59
**18**	1	1	0	0.3	0	0	2	0	0.02	0.0038	7.67	0.69	8.14
**19**	1	1	0.5	0.3	0.2	0.2	2	0.1	0.02	0.0038	7.94	0.47	9.27
**BLOCK 2**													
20*	0.5	0.5	0.25	0.15	0.1	0.1	1.25	0.05	0.01	0.0019	7.60	0.49	4.23
21*	0.5	0.5	0.25	0.15	0.1	0.1	1.25	0.05	0.01	0.0019	7.79	0.50	4.23
**22**	1	0	0.5	0	0	0.2	2	0	0.02	0	6.16	0.38	4.49
**23**	1	0	0.5	0	0.2	0.2	0.5	0.1	0	0.0038	7.46	0.35	4.72
**24**	0	1	0.5	0.3	0.2	0	0.5	0	0	0.0038	7.66	0.44	4.53
**25**	1	0	0	0	0.2	0	2	0.1	0.02	0	7.56	0.91	3.81
**26**	1	0	0.5	0.3	0.2	0	0.5	0	0.02	0	7.69	0.21	4.53
**27**	1	0	0	0.3	0	0.2	0.5	0.1	0.02	0	7.66	0.21	4.10
**28**	0	1	0	0.3	0	0.2	0.5	0.1	0	0.0038	7.67	0.20	4.10
**29**	0	1	0.5	0	0	0.2	2	0	0	0.0038	7.58	0.31	4.49
30*	0.5	0.5	0.25	0.15	0.1	0.1	1.25	0.05	0.01	0.0019	7.82	0.43	4.23
**31**	0	1	0	0	0	0	0.5	0	0.02	0	7.10	1.17	3.59
**32**	1	0	0.5	0.3	0	0	2	0.1	0	0.0038	7.83	0.61	4.35
**33**	1	0	0	0.3	0.2	0.2	2	0	0	0.0038	7.64	0.30	4.27
**34**	0	1	0.5	0.3	0	0	2	0.1	0.02	0	7.72	0.51	4.35
**35**	0	1	0	0.3	0.2	0.2	2	0	0.02	0	7.74	0.30	4.27
**36**	1	0	0	0	0	0	0.5	0	0	0.0038	7.27	1.36	3.59
**37**	0	1	0.5	0	0.2	0.2	0.5	0.1	0.02	0	7.84	0.40	4.72
**38**	0	1	0	0	0.2	0	2	0.1	0	0.0038	7.68	0.60	3.81

Factors: **MP**: meat peptone; **ME**: meat extract; **YE**: yeast extract; **SA**: sodium acetate; **K**: K_2_HPO_4_; **AC**: ammonium citrate; **G**: glucose; **T**: Tween 80; **Mg**: MgSO_4_.7H_2_O; **Mn**: MnSO_4_.H_2_O.

*central points,

^♯^pH (pH_initial_−pH_final_)

Later, we evaluated the growth of *L*. *reuteri* CRL1098 in an optimized culture medium. In addition, the effect of agitation and the ability of this biomass to produce LrS in RPMI 1640 medium were also determined. The batch fermentation experiments were carried out in formulated culture medium (named PETG medium: 0.5% meat peptone, 0.5% meat extract, 0.1% Tween 80 and 0.5% glucose). *L*. *reuteri* CRL1098 was inoculated (2% v/v) in 100 mL (Pyrex bottles 100 mL) and incubated at 37°C with and without agitation (rotary shaker at 150 rpm, Marconi, Piracicaba, Brazil)for 3h. Batch culture experiments were performed under uncontrolled pH. Both cultures were centrifuged (8,000 g for 10 min), washed with phosphate-buffered saline (PBS) and resuspended (7.5 LogCFU/mL) in RPMI 1640 medium (GIBCO, Grand Island, NY, USA). These bacterial suspensions were incubated for 4h at 37°C in agitating conditions (rotary shaker at 150 rpm). After, the suspensions were centrifuged and LrS was obtained by aseptic filtration using 0.22 mm pore size low protein binding cellulose acetate filters (Millipore, Bedford, MA, USA). LrS was frozen at -20°C for further experiments. The anti-inflammatory activity of LrS was determined as described below.

On an industrial scale, the lactic cultures are produced in bioreactors and harvested in stationary phase when the maximum biomass production is reached. Therefore, the *L*. *reuteri* CRL1098 biomass production in bioreactors and the obtaining of LrS in RPMI 1640 medium were evaluated. To accomplish this, batch fermentations were performed in a 2 L bioreactor (INFORS HT, Switzerland). The PETG medium (1.5 L, pH 6.5) was added to the bioreactor and inoculated with *L*. *reuteri* CRL 1098 cultures (2%, v/v). During 24h fermentation, the temperature was maintained at 37 °C, the agitation speed at 150 rpm. Batch cultures experiments were performed under controlled and uncontrolled pH. When the controlled pH experiment was conducted, pH was maintained at 5.5 by adding 2 M NaOH (pH value used to produce biomass of *Lactobacillus* in our laboratory). Samples were withdrawn periodically to determine cell growth (Log CFU/mL), acidification (pH) and glucose consumption. pH measurements were determined with a digital pH meter (Altronix TPX 1, New York, USA).

In order to evaluate the effect of growth stages on the ability of the microorganism to produce LrS, the fermentation time in bioreactors was extended until to 24h. Cells obtained from free and controlled pH cultures in different growth stages were centrifuged (8,000 g for 10 min), washed with phosphate-buffered saline (PBS), resuspended (7.5 Log CFU/mL) in RPMI 1640 medium and incubated for 4h at 37°C in agitating conditions (rotary shaker at 150 rpm). The bacterial suspension was centrifuged and LrS was aseptically filtered. The anti-inflammatory activity of LrS was determined as described below.

### Second step: Formulation of a low-cost medium for anti-inflammatory LrS production

In the second step, *L*. *reuteri* CRL1098 biomass is resuspended in RPMI 1640 medium and incubated for 4h (Fig 1). Under these experimental conditions, the microorganism was capable of living but did not proliferate. In order to reduce the cost of RPMI medium, different media and buffer solutions were tested.

The batch fermentation experiments were carried out in PETG medium with agitation (150rpm), under uncontrolled pH condition during 6 h at 37°C (optimized conditions to biomass production in previous point). Culture were centrifuged (8,000 g for 10 min), washed with phosphate-buffered saline (PBS) and, resuspended in the following media and solutions: RPMI 1640 medium (control), DMEM medium (GIBCO, Grand Island, NY, USA), Bacto^™^Casitone medium (BD Biosciences. Becton, Dickinson and Company. CA, USA), phosphate buffer saline (PBS), tris-(hydroxymethyl)-aminomethane (Tris buffer) and sodium citrate buffer with and without glucose. All these media and solutions were later assayed. The bacterial suspensions were incubated for 4h at 37°C in agitating conditions (rotary shaker at 150 rpm); then they were centrifuged and aseptically filtered to obtain LrS. The anti-inflammatory activity of LrS was determined as described below.

### Anti-inflammatory activity of LrS

To study the anti-inflammatory activity of LrS, mouse macrophage cell line RAW 264.7 was cultured in DMEM supplemented with 10% fetal bovine serum (FBS) (NATOCOR, Córdoba, Argentina), 100 IU/mL penicillin and 100 mg/mL streptomycin and cultured at 37°C in a 5% CO_2_ incubator. Prior to the addition of LrS, cells were seeded in 24-well culture plates, lowed to adhere for 8h at 37 °C in 5% CO_2_ atmosphere. Once LrS was added, the cells were incubated for 4h. Finally, LPS (100 ng/mL final concentration) from *Escherichia (E*.*) coli* serotype O26: B6 (Sigma, St. Louis, MO, USA) was added to cultures and incubated for 20 h. Different anti-inflammatory parameters were determined. Nitric oxide (NO) concentrations in supernatants of RAW 264.7 cells were determined by using Griess reagent (Promega Corporation, Madison, WI, USA). 50 mL aliquots of medium were mixed with an equal volume of sulfanilamide solution (1% sulfanilamide in 5% phosphoric acid) at different times and left to stand for 10 min at room temperature in the dark. Then, 50 mL of NED solution [0.1% N-(1-Naphthyl) ethylenediamine] were added and left to stand for 10 min at room temperature in the dark. The absorbance was measured at 540nm with a microplate reader (VERSAmax, Molecular devices, Sunnyvale, CA, USA). Standard curve for sodium nitrite was used to acquire the level of NO produced by treated RAW 264.7 cells.

TNF-α concentration was measured in cell free supernatants of RAW 264.7 cells using enzyme-linked immunosorbent assay kits (ELISA Ready-Set- Go!eBioscience, San Diego, CA, USA).

### Statistical data analysis

All assays were performed at least in triplicate and the results were expressed as mean values with standard deviations. Statistical analyses were performed using MINITAB 14 software (State College, PA, USA). Comparisons were accomplished by ANOVA general linear model followed by Fisher test. Statistically significant differences were defined at a *p* value <0.05.

## Results

### First step: Design of a low-cost culture medium for *L*. *reuteri* CRL1098 biomass production and evaluation of their functional property

The first step on the search for optimal conditions for biomass production was to identify the variables with significant influence. The statistical design resulted into 38 culture media defined in 2 blocks with three central points in each block (Table 1). Table 1 summarizes *L*. *reuteri* CRL1098 growth (Log CFU/mL), ΔpH (pH_initial_−pH_final_) and the cost of each medium assayed. In most culture medium, the bacterial growth reached approximately 7.4–7.9Log CFU/mL and significant changes in pH were detected (ΔpH 0.47 to 0.75). Remarkably, the higher acidification was detected (ΔpH 1.62) in medium number 4; however, a low growth (7.13 Log CFU/mL) was observed. The cost of each one of the media evaluated was different, varying from 0.59 to 9.27 USD/L.

Three components of the culture medium; meat peptone, meat extract and Tween 80, showed a positive effect on bacterial growth (Table 2). In addition, using this statistical design, interaction effects among factors on the biomass production evidenced positive and negative interactions (S1 Fig). The most important positive interaction occurred among meat peptone and meat extract: an increase of meat peptone and meat extract concentration (from 0 to 1%) improved *L*. *reuteri* growth. However, the intermediate value (0.5%) showed a similar growth to the maximum concentration (1%). On the other hand, a positive effect of Tween 80 was observed with the highest concentration (0.1%).

**Table 2:** Predicted effects of the factors on the growth of *L*. *reuteri* CRL1098.

Factors	Effect	Coefficient	*p*-Value
**Meat peptone**	0.13655	0.02963	0.004*
**Meat extract**	0.19645	0.13687	0.001*
**Yeast extract**	0.00125	-0.03802	0.965
**Sodium acetate**	0.02673	0.05201	0.366
**K**_**2**_**HPO**_**4**_	-0.02422	0.02653	0.409
**Ammonium citrate**	-0.03422	-0.05575	0.259
**Glucose**	0.00437	0.03646	0.877
**Tween 80**	0.11259	0.09494	0.009*
**MgSO**_**4**_.**7H**_**2**_**O**	0.04415	-0.01657	0.162
**MnSO**_**4**_.**H**_**2**_**O**	0.00651	0.04190	0.818

*Significantly different effect (*p*<0.05).

According to significant effect and interactions, the composition of the culture medium, named PETGculture medium, was: an intermediate concentration (0.5%) of meat peptone and meat extract, and a high concentration (0.1%) of Tween 80. Glucose was added at a minimal concentration (0.5%) as a carbon source. To validate this medium, *L*. *reuteri* CRL1098 was cultured both in MRS and PETG culture media for 3 h without agitation. *L*. *reuteri* CRL1098 growth in PETG culture medium, was similar to that observed in MRS medium (7.9 ± 0.02 Log CFU/mL), Thus, PETG culture medium could replace MRS medium and its cost was only 3.81 USD/L compared with 9.27 USD/L of MRS medium (Fig 1).

**Fig 2:**
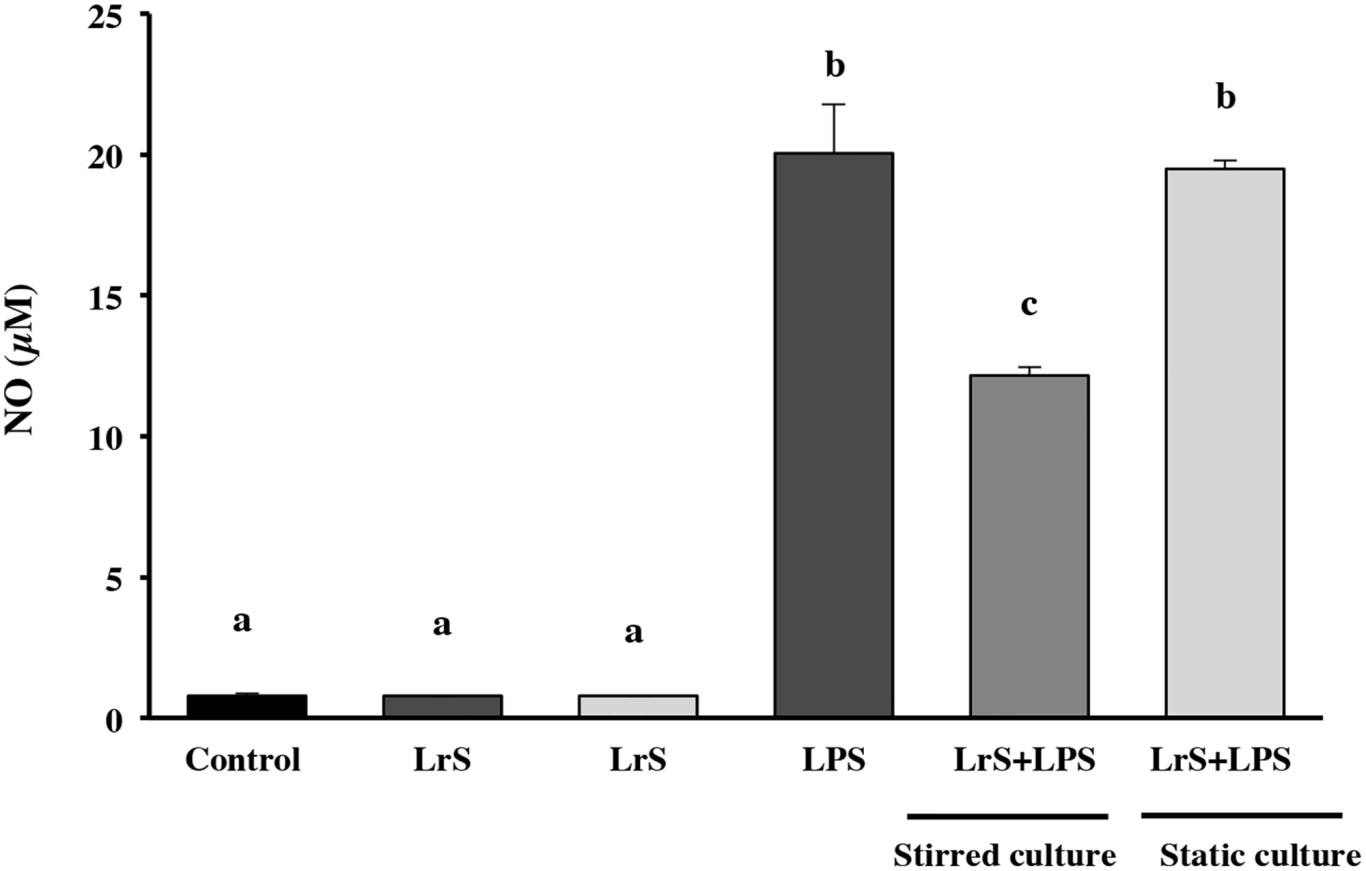
Effect of LrS on NO production by RAW 264.7 macrophages. LrS production by bacteria grown in under static and stirred conditions was evaluated in RPMI 1640 medium, and their anti-inflammatory effect was checked on LPS challenged RAW cells. NO was measured in culture supernatants by Griess assay. Data are representative result of three independent experiments. Values not sharing the same letter were significantly different (*p*-value<0.05).

The culture conditions could affect the biomass yield as well as the functional properties of *Lactobacillus*. Consequently, the current study evaluated different factors, such as culture medium, agitation, pH and growth phases on biomass production and anti-inflammatory activity of LrS obtained in RPMI 1640 medium. First, the effect of agitation on biomass production was evaluated. Batch experiments were carried out in PETG culture medium with or without agitation for 3h under uncontrolled pH at 37°C. *L*. *reuteri* CRL1098 growth was similar in both conditions (7.6 ± 0.2 Log CFU/mL). Subsequently, the production on LrS by bacteria grown in both conditions was evaluated in RPMI 1640 medium, and their anti-inflammatory effect was checked on LPS challenged RAW cells. Stimulation of cells with LPS significantly increased NO production by RAW 264.7 cells (Fig 2). The levels of NO were significantly lower (35%) in LPS-challenged cells treated with LrS produced by *L*. *reuteri* grown under stirring conditions. However, this effect was not induced by the LrS produced by the microorganism grown under static conditions: the levels of NO were the same than the ones observed in LPS-challenged cells (Fig 2).

**Fig 3:**
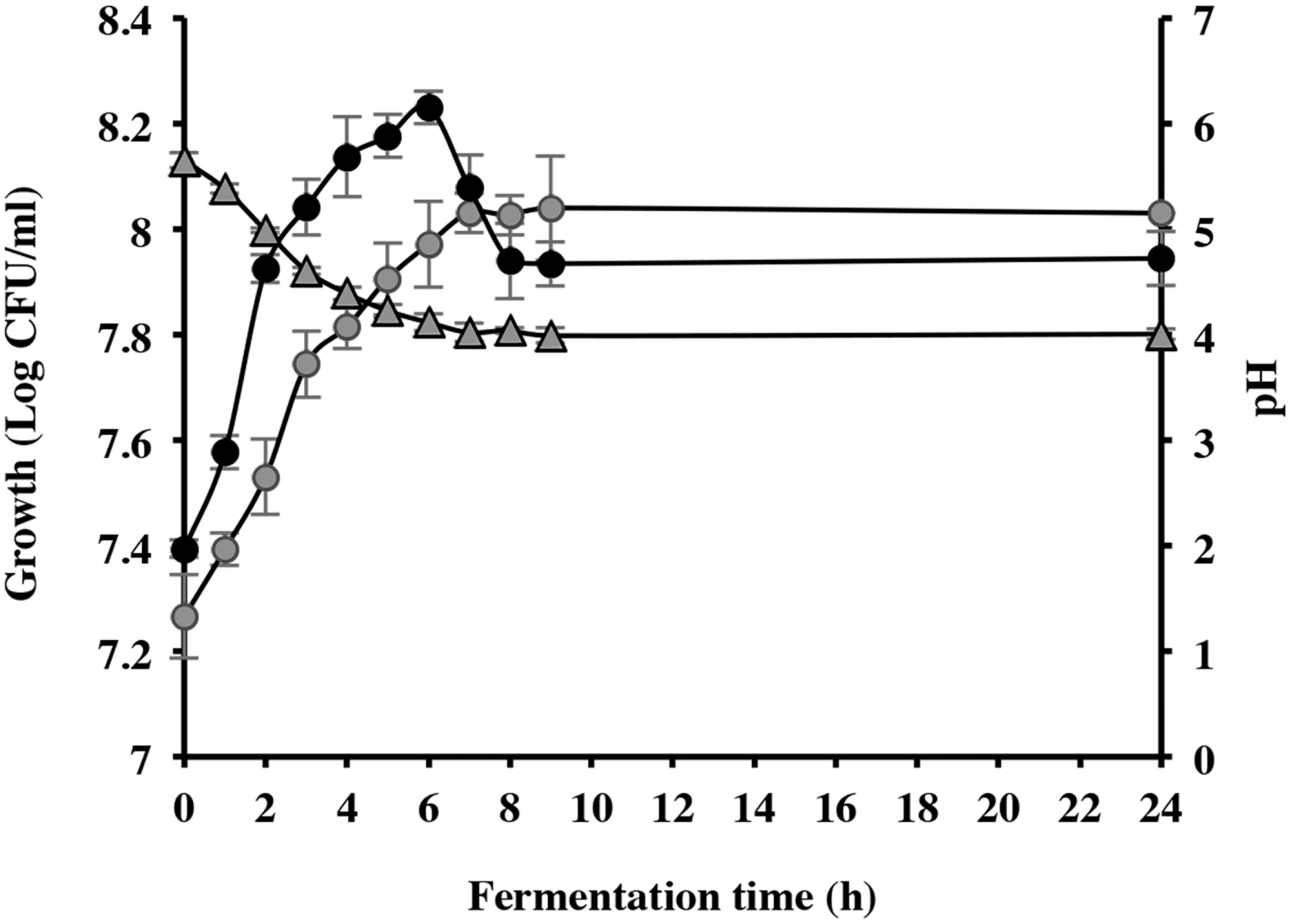
*L*. *reuteri* CRL1098 growth in PETG medium under controlled and uncontrolled pH. (black circle) pH-controlled culture (gray circle) pH-uncontrolled culture; (gray triangle) pH.

Considering that pH plays a key role in growth, its effect was evaluated in a laboratory bioreactor (1.5 l) with continuous agitation (150 rpm) under controlled and uncontrolled pH for 24 h (Fig 3). The cultures under uncontrolled pH conditions reached stationary growth phase at 7 h incubation (8.0 ± 0.2 Log CFU/mL) (Fig 3). These values were achieved earlier (3h) at controlled pH conditions. However, although pH was controlled, a significantly viability decrease was observed after 6h of fermentation (Fig 3), probably because glucose was rapidly and almost completely consumed: from 5.0 to 0.9 g/L at 6h, and only 0.27 g/L of glucose was detected at 24h.

**Fig 4:**
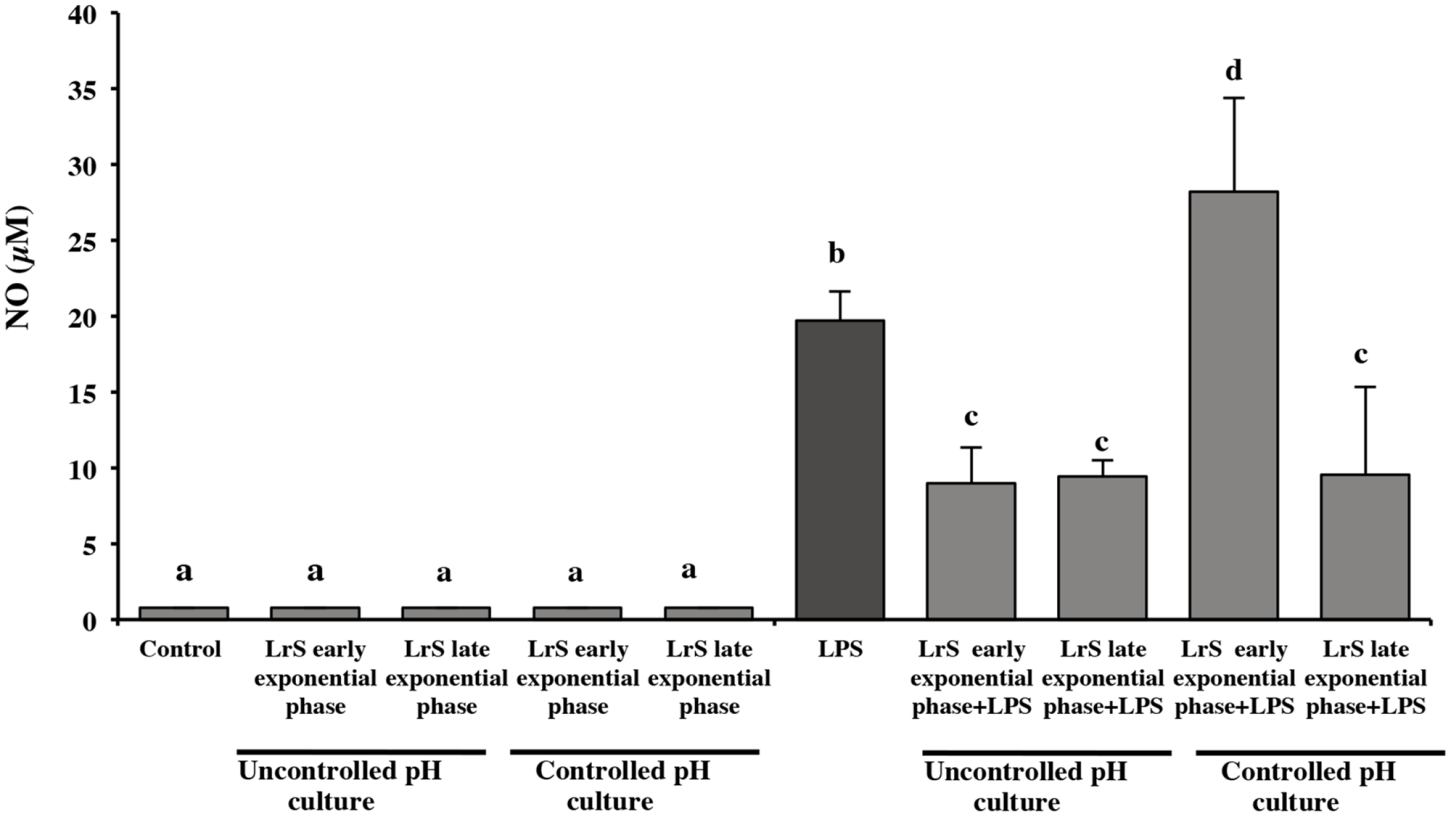
Effect of LrS obtained in RPMI medium on NO production by RAW 264.7 macrophages. RAW 264.7 cells were challenged with LPS and LrS obtained from bacteria grow in PETG medium in early and late exponential phase, at controlled and uncontrolled pH conditions. Cells with a without LPS challenge were used as control. NO production was measured in culture supernatants by Griess assay. Data shown are a representative result of three independent experiments. Values not sharing the same letter were significantly different (p-value<0.05).

On an industrial scale, the lactic cultures are harvested in stationary phase when the maximum biomass production is reached. However, previous studies in our laboratory demonstrated that *L*. *reuteri* CRL 1098 in exponential growth phase (MRS medium, uncontrolled pH) was able to produce active LrS (reduction TNF-α production in PBMC) [18] **(**Fig 1). With this approach, the ability of bacterial cells at different phases of growth, obtained from controlled and uncontrolled pH cultures (early exponential, late exponential and stationary phases) to produce anti-inflammatory LrS in RPMI 1640 medium was evaluated. At uncontrolled pH, the anti-inflammatory activity of LrS obtained from both growth phase, early and late exponential was similar (Fig 4). On the contrary, different results were observed in controlled pH cultures, in which the anti-inflammatory capacity of LrS depended on the growth phase of the microorganism. The anti-inflammatory activity was only observed in LrS obtained from biomass in late exponential phase. Regardless the pH conditions of the cultures, the LrS obtained from the biomass at the stationary phase showed a high cytotoxicity on RAW 264.7 cells (data not shown).

**Fig 5:**
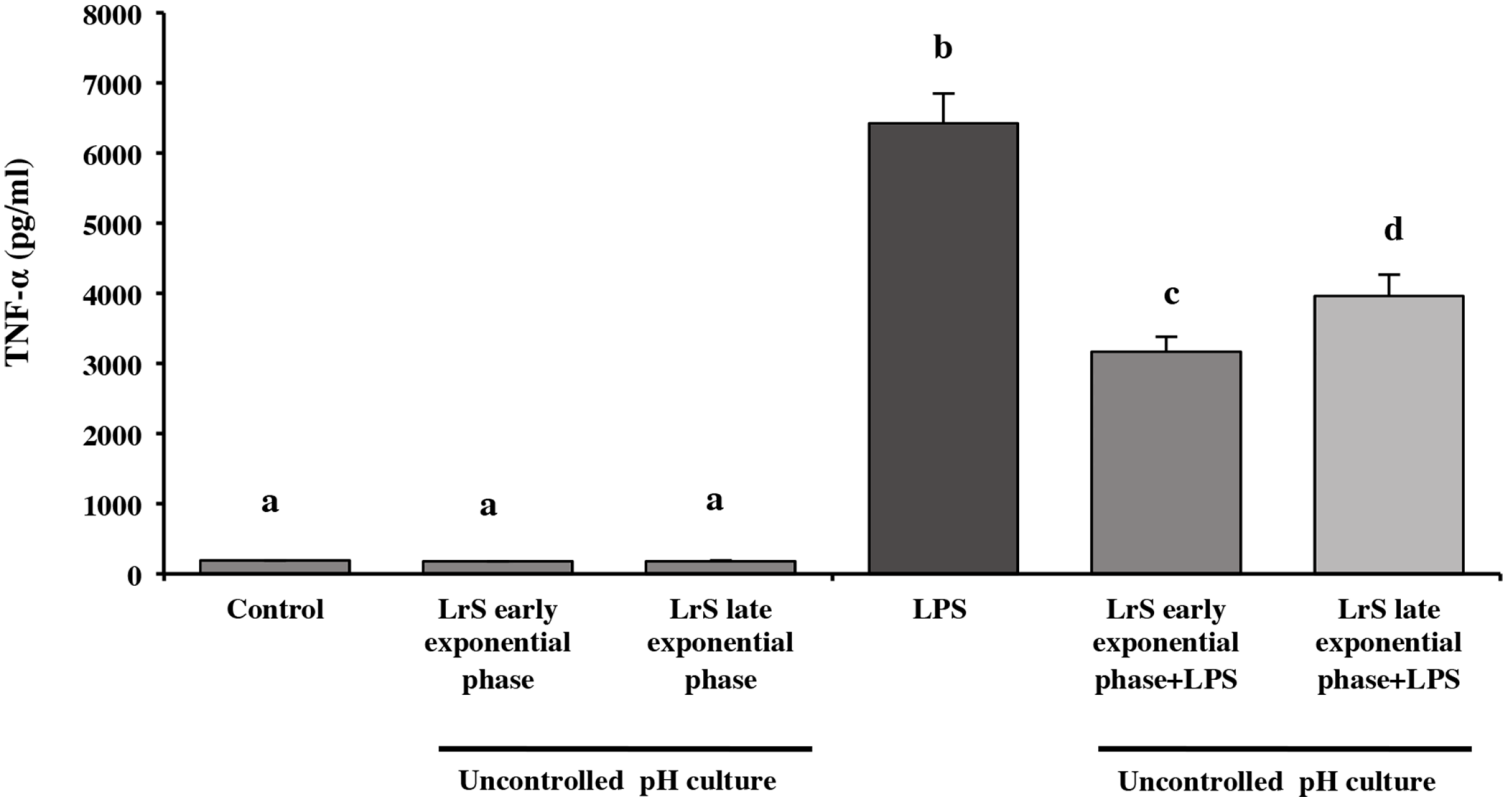
Effect of LrS on TNF-α production by RAW 264.7 cells. LrS production by bacteria grow in PETG medium in early and late exponential growth phase was evaluated in RPMI 1640 medium, and their anti-inflammatory effect was checked on LPS challenged RAW cells. TNF-α production was measured in culture supernatants by ELISA. Data shown are a representative result of three independent experiments. Values not sharing the same letter were significantly different (*p*-value<0.05).

When the production of TNF-α was evaluated as a marker of inflammation, it was observed that the LrS obtained from uncontrolled pH cultures in both early and late exponential phases, showed a significant decrease (*p* ≤ 0.05) of this marker (50 and 38%, respectively) regarding the control of cells stimulated with LPS (Fig 5).

The quantification of the two inflammatory markers (NO and TNF-α) allowed us to optimize the first stepof biomass production (Fig 1) establishing the conditions of *L*. *reuteri* CRL1098 culture: PETG medium, uncontrolled pH, agitation (150 rpm), 37°C and harvest of the biomass in late exponential growth phase (6h of incubation).

### Second step: Formulation of a low-cost medium for anti-inflammatory LrS production

In a second step, we aimed to formulate a low cost medium for LrS production. Previously, LrS was obtained after 4h incubation time of *L*. *reuteri* CRL1098 in RPMI 1640 medium(129 USD/L) in our laboratory (Fig 1). In these conditions, the microorganism was able live but did not proliferate.

Considering the results of the previous point, *L*. *reuteri* CRL1098 biomass was obtained from PETG culture medium in optimized conditions. Subsequently, this biomass was suspended in different media and buffers. Bacteria survival assay in different media and buffers was performed during 4h at 37 °C under agitated condition. Results indicated that *L*. *reuteri* CRL1098 only lived, but with a non-proliferating condition in casitone medium (1%) and sodium citrate buffer with 4 g/L glucose as carbon source (Table 3).

**Table 3:** Viability of *L*. *reuteri* CRL1098 in different media after 4h at 37 °C under agitating condition.

Media	Growth (Log CFU/mL)		
	0 h	2 h	4 h
**RPMI 1640**	7.60 ± 0.08	7.44 ± 0.07	7.40 ± 0.25
**DMEM**	7.75 ± 0.03	6.49 ± 0.08	5.48 ± 0.10
**Casitone 1%**	7.77 ± 0.05	7.72 ± 0.05	7.61 ± 0.12
**Casitone 1:10**	7.44 ± 0.02	7.19 ± 0.05	6.73 ± 0.01
**Casitone 1:20**	7.53 ± 0.02	7.23 ± 0.04	6.83 ± 0.06
**Casitone + Glucose 4 (g/L)**	7.56 ± 0.04	7.83 ± 0.05	8.30 ± 0.13
**Tris buffer + Glucose 4 (g/L)**	7.64 ± 0.03	7.56 ± 0.01	6.45 ± 0.04
**Sodium citrate buffer + Glucose 4 (g/L)**	7.62 ± 0.10	7.63 ± 0.04	7.69 ± 0.07
**PBS buffer + Glucose 4 (g/L)**	7.76 ± 0.13	7.28 ± 0.08	6.52 ± 0.06

**Fig 6:**
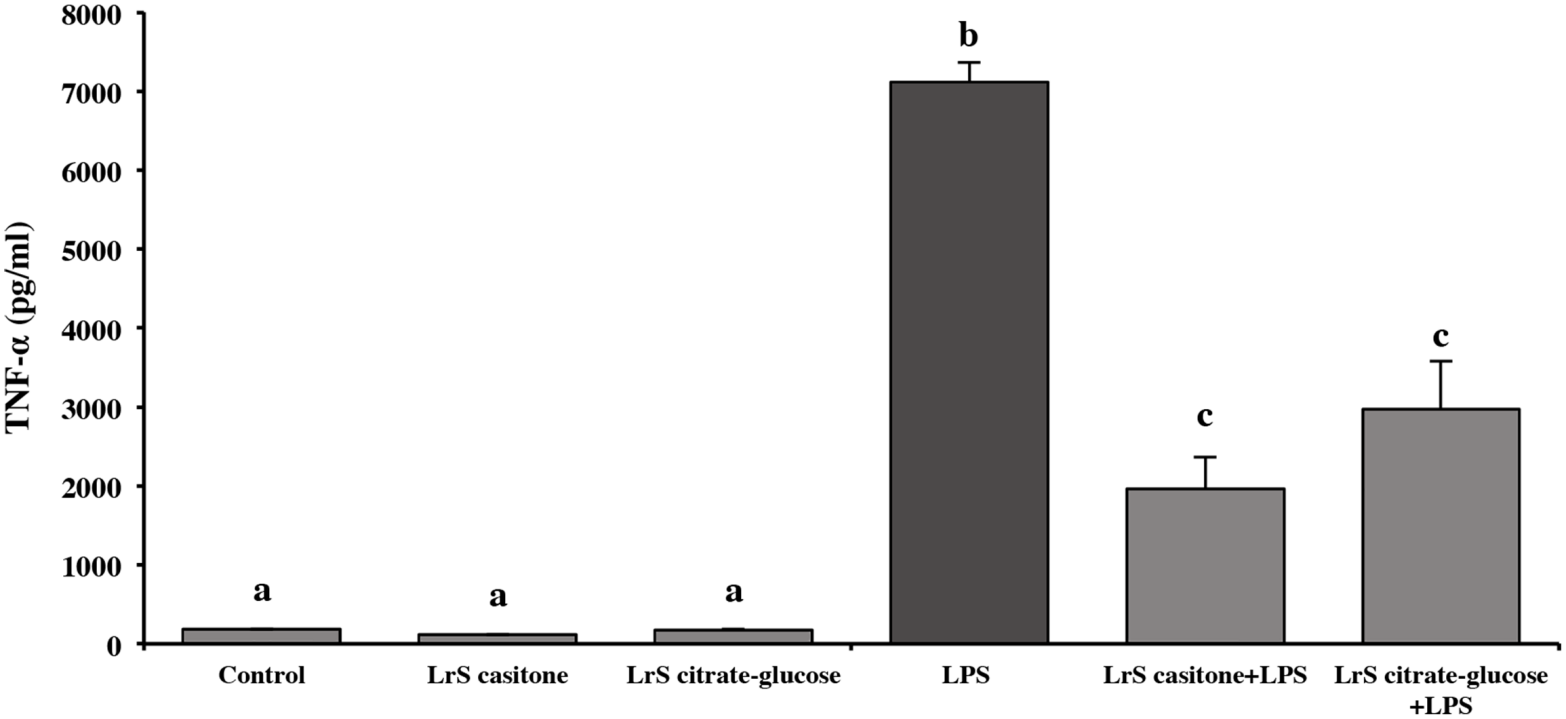
Effect of LrS on TNF-α production by RAW 264.7 macrophages. LrS production by bacteria grow in PETG medium (late exponential growth phase, pH-uncontrolled culture) was evaluated in casitone medium and sodium citrate buffer, and their anti-inflammatory effect was checked on LPS challenged RAW cells. TNF-α production was measured in culture supernatants. Data shown are a representative result of three independent experiments. Values not sharing the same letter were significantly different (*p*-value<0.05).

To evaluate anti-inflammatory activity of LrS obtained from casitone medium and citrate buffer, LPS stimulates RAW 264.7 cells were treated with both LrS. TNF-α production was determined in culture supernatant (Fig 6). Both LrS diminished TNF-α: 72% diminution induced by LrS obtained in casitone medium and 61% by LrS obtained in citrate buffer.

Casitone medium (4.39 USD/L) is hydrolyzed casein composed of a large percentage of peptides that could interfere in the determination of anti-inflammatory activity. For these reasons, citrate buffer was selected as the optimal means for producing the anti-inflammatory compound. It is a simple and economical buffer (2.37 USD/L) that allows the obtention of an acceptable level of anti-inflammatory activity.

## Discussion

Research reports revealed that certain extracellular peptides produced by probiotic bacteria are responsible for some of their beneficial effects [21–22]. In these works, culture supernatant containing the functional peptides is used, not the purified peptides, despite the fact that its amino acid sequence is available [21–25]. In addition, it has been found that the effect of culture supernatant or purified peptides is similar. Thus, this point would be of great industrial importance since aculture supernatant could be used and whose production is of lower cost than a purified peptide production obtained with the use of heterologous strain to overexpress this peptide. On the other hand, the chemical synthesis of peptides to a large scale has a high-cost [26]. We have demonstrated previously that a LrS produced by *L*.*reuteri* CRL1098 modulates the inflammatory response *in vitro* and in a mice model. In this work, we propose an economic process of LrS realized through two steps: biomass and LrS production.

Regarding the first step, usually the probiotic LAB production involves batch growth in large-scale bioreactors. LAB batch culture has been extensively studied before [27–30]. These studies evaluate the application of economic and more competitive culture media for biomass production, because the growth medium is the most expensive factorat an industrial level [31]. Statistical models are often used for simplified media formulation, through the exclusion or reduction of components of the medium [15, 32, 33]. The initial approach involves the use of a statistical model, employing the conventional laboratory medium MRS. MRS broth remains the most widely used culture medium for LABgrowth [34]. However, it is too expensive for industrial applications [15]. In this work, MRS medium is used for biomass production in the first step of the studied process. The experiments were performed with different combinations of ten MRS factors to determine their impact on biomass production by using a fractional factorial design. The statistical design allowed us to reduce the number of experimental runs, to identify the important factors, and to determine the interaction between factors without losing the information on the main effects and their interactions [35]. Among the ten factors studied, only meat peptone, meat extract and Tween 80 had significant influence on the growth of *L*. *reuteri* CRL1098. Therefore, these components were required for optimal biomass production. Several studies have reported the nutrients influence on LAB biomass production, mainly the positive effect of nitrogen sources, such as, peptone and meat extract. Protein hydrolyzates are widely used in LAB growth culture and account for 70% of the total cost of medium [31, 36]. However, different authors reported the strong effect of nitrogen sources on the growth of *Lactobacillus* strains, such as, *L*. *casei* KH-1 [37], *L*. *acidophilus* FTCC 0291 [35], *L*. *rhamnosus* E/N [33] and *L*. *reuteri* [38]. Hebert et al. [39] showed that Tween 80 had different effects on the growth of several LAB: it has stimulated *L*. *delbrueckii* subsp. *bulgaricus* NCFB 2772 and has inhibited *L*. *sake* NCFB 2714. On the contrary, this compound was essential for *L*. *curvatus* [40] and had a great positive effect on the growth of *L*. *reuteri* [39]. Our results showed a positive curve slope indicating a strong effect of Tween 80 on *L*. *reuteri* CRL1098 biomass production. According to statistical design (Table 2), many components in MRS medium, such as, sodium acetate, yeast extract, K_2_HPO_4_, ammonium citrate, glucose, MgSO_4_.7H_2_O and MnSO_4_.H_2_O, were considered not to have a significant effect on the biomass production in the assay conditions. Therefore, these components could be removed from the medium without influencing bacterium growth. This was reported by Mataragas et al. [41] for *Leuconostoc mesenteroides* L124 and *L*. *curvatus* L442, having similar results. Our present results showed the predicted optimal components concentration: 0.5% meat peptone, 0.5% meat extract, 0.1% Tween 80 and 0.5% glucose. In view of these optimal components a PETG culture medium was designed, one sustained *L*. *reuteri* CRL 1098 growth. In addition, LrS from PETG culture medium showed the anti-inflammatory effect on LPS stimulated RAW 264.7 cells. The analysis of costs indicated that the PETG culture medium price is 3.81 USD/L, 2.4 times lower than MRS medium cost (9.27 USD/L).

In this study, the simultaneous evaluation of the effect of different factors on the growth and LrS production of a *Lactobacilus* strain was performed. Biomass production was found to depend mainly on culture medium, while LrS production with anti-inflammatory activity depended of culture conditions such as pH, agitation and growth phase. Previous studies showed that fermentation parameters and culture conditions affect the biomass yield as well as the functional properties of *Lactobacillus* [42, 43]. Aguirre-Ezkauriatza et al. [44] reported that under agitating conditions, higher biomass was acquired as compared to static condition, because agitation enhances microbial growth by improving the distribution of nutrients through the fermentation tank. In our study, we found similar *L*. *reuteri* CRL1098 growth under static and agitating conditions. Nevertheless, the best anti-inflammatory response was observed in cell free supernatant obtained from bacterial culture under agitating condition. *L*. *reuteri* is considered an oxygen-tolerant anaerobe with fermentative metabolism. The growth condition and the type of metabolism significantly affect the stress responses in LAB. Several studies have demonstrated that conditions which promote aerobic and respiratory growth (agitation) induce stress tolerance in *Lactobacillus* [45]. So, this growth condition could also have influenced the ability of *L*. *reuteri* to produce active LrS (second step, under cell starvation conditions).

Other important parameter for cultivating LAB is the pH of the fermentation [11]. Numerous studies indicate that the medium pH can influence the microorganisms´ growth, affecting cellular functions and metabolic processes [46, 47]. Rault et al. [48] showed that the pH influenced the growth behavior of lactobacilli and the final yield obtained, although substantial differences can be observed, depending on the strain. The control of fermentation pH has also been used to improve metabolite production of LAB [41, 49, 50]. The optimum pH range for the growth of LAB varies from 3.5 to 6.5 [51]. LeBlanc et al. [52]observed a similar bacterial growth of *Lactobacillus* strains at different pH conditions of the fermentation medium. However, our experiment showed a significant viability decrease after 6h of fermentation under controlled pH; this could be explained by the fast glucose consumption (82% at 6 h).

Probiotic bacteria at an industrial level are commonly harvested during the stationary growth phase to ensure the highest biomass production [53]. In the present work, the LrS obtained from *L*. *reuteri* CRL1098 harvested at stationary phase in both controlled and uncontrolled pH conditions showed a high cytotoxicity on RAW 264.7 cells. This could be due to the production of some toxic metabolite during this growth phase. Some works reported that the growth phase during which probiotics are harvested can influence their capability as health-promoting agents [53]. Deepika et al. [54] reported that cell surface properties of *L*. *rhamnosus* GG change during the transition from the exponential to the stationary growth phase, affecting the ability of the strain to adhere to epithelial cells. *Lactobacillus* strains can differentially affect antigen-specific antibody subclasses IgG1 and IgG2a, dependent on the growth phase. According to Maassen et al. [55], this differential antibody response is due to growth phase-dependent differences in bacterial cell wall composition. Proteins secreted by probiotic LAB at early stationary growth phase were reported in other works; however, they are produced in MRS medium [56, 57].

At laboratory scale LrSproduction (second step) was produced only under stress conditions in RPMI 1640 medium, where the bacteria was able to live but did not proliferate [18]. Some preliminary assays revealed that when the LrS production medium (RPMI medium) was modified by increasing glucose or by adding some essential components (adenine, alanine, glutamine or Tween 80), *L*. *reuteri* CRL 1098 grew but the supernatant lost the anti-inflammatory effect. These results showed that the compound is not associated with the growth of *L*. *reuteri* (unpublished data). Also, the RPMI 1640 medium used in the second step of LrS production is very expensive (129 USD/L). Therefore, the design of an economic and more competitive medium for LrS production represented an important step for its application at an industrial scale. In order tochange the medium for LrS production for industrial uses, we evaluated the effect of economic media and buffer solutions. Up to date, we did not find reports about culture media optimization for LAB peptides production under non-proliferating conditions. In contrast, several studies reported culture media optimization for bacteriocins production by LAB, antimicrobial peptides which synthesis is associated with bacteria growth [15, 58–60]. In this work,sodiumcitrate buffer with glucose (2.37 USD/L) was selected for the anti-inflammatory compound production. Moreover, LrS obtained in sodium citrate buffer had an anti-inflammatory activity similar from that obtained in RPMI 1640 medium. Farias and Manca de Nadraet al. [61] reported that *Oenococcus oeni* showed extracellular proteolytic activity with exoprotease production when subjected to a total energy and nutrient starvation regime (2 h of incubation in citrate buffer). The production of metabolites by starved bacterial suggests that it could be an effective mechanism for survival in the stress conditions encountered during starvation.

## Conclusions

Based on the studies presented in this work, the two-step production of the anti-inflammatory compound by *L*. *reuteri* CRL1098 was optimized. Our results suggest that the fermentation procedures are important for the industrial production of probiotics or its active metabolites. The cost analysis reveals the importance of the obtained results: 6.18 USD/L is the cost of producing the LrS with anti-inflammatory activity in the optimized process (PETG culture medium: 3.81 USD/L, citrate buffer: 2.37 USD/L), compared to 138 USD/L price that responds to the original laboratory scale process (Fig 1). In this analysis, only the item supplies (culture media, reagents, drugs) were considered for its impact on the production process, as a starting point for its optimization. The overall results demonstrated that the strategies applied allowed a 95% cost reduction from the original laboratory-scale process.

## Supporting information

S1 Fig Graph of factor interaction as a function o*f L*. *reuteri* CRL1098 growth. MP: meat peptone; ME: meat extract; YE: yeast extract; SA: sodium acetate; K: K_2_HPO_4_; AC: ammonium citrate; G: glucose; T: Tween 80; Mg: MgSO_4_.7H_2_O; Mn: MnSO_4_.H_2_O.
(TIF)
