# Supplementary figures and images for: Two-step production of anti-inflammatory soluble factor by Lactobacillus reuteri CRL 1098
Source: PLoS One. 2018 Jul 6;13(7):e0200426. doi: 10.1371/journal.pone.0200426 (PMC6034873; doi:10.1371/journal.pone.0200426)

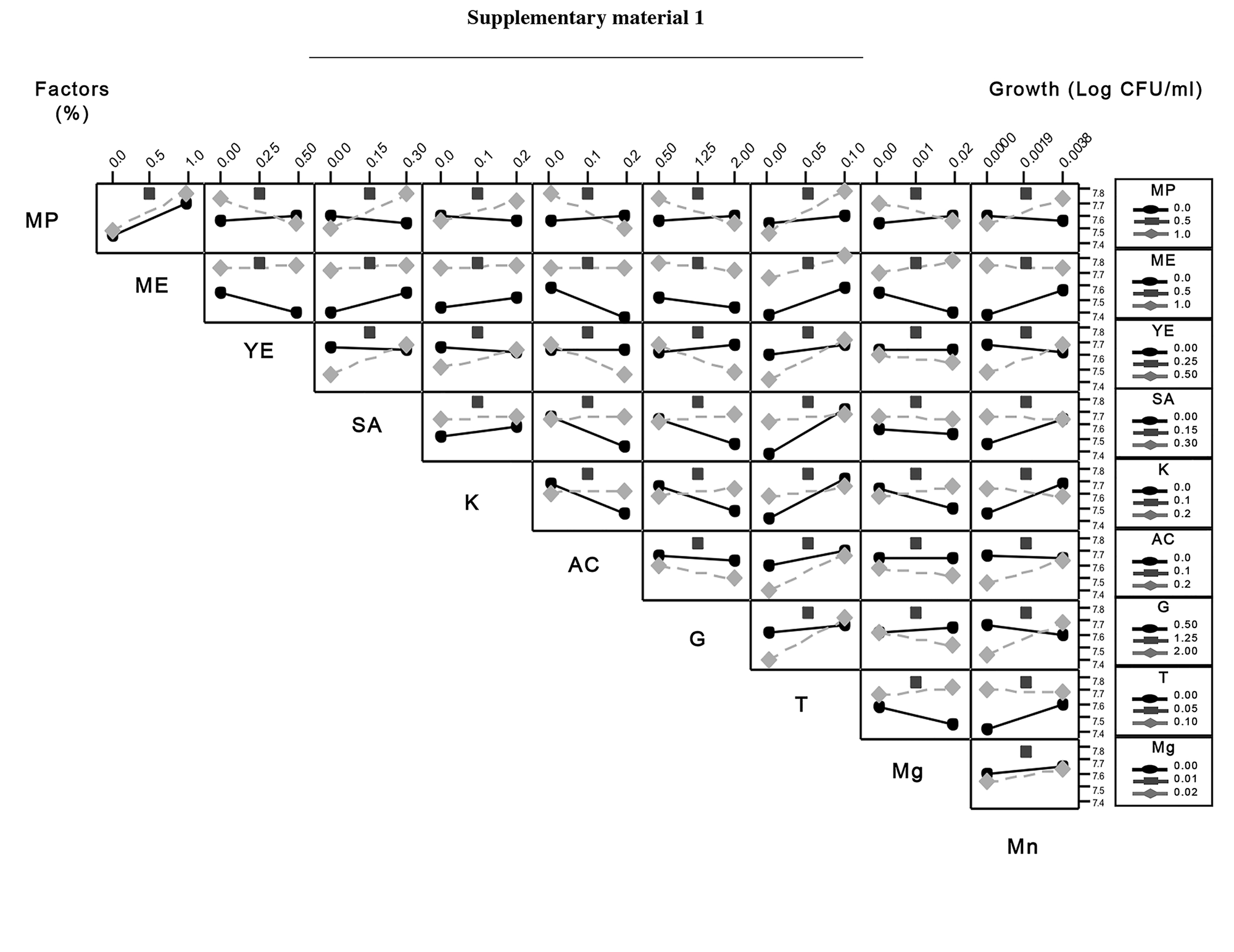

Supplement: S1 Fig — MP: meat peptone; ME: meat extract; YE: yeast extract; SA: sodium acetate; K: K2HPO4; AC: ammonium citrate; G: glucose; T: Tween 80; Mg: MgSO4.7H2O; Mn: MnSO4.H2O. (TIF) [file pone.0200426.s001.tif]
